# Successful treatment and remission of advanced testicular cancer after COVID‐19 infection during induction chemotherapy

**DOI:** 10.1002/iju5.12726

**Published:** 2024-05-01

**Authors:** Goshi Kitano, Shiori Tanaka, Manabu Kato, Naoya Itoh, Takahiro Kojima

**Affiliations:** ^1^ Department of Urology Aichi Cancer Center Hospital Nagoya Aichi Japan; ^2^ Deparment of Infectious Diseases Aichi Cancer Center Hospital Nagoya Aichi Japan

**Keywords:** anticancer chemotherapy, COVID‐19, embryonal carcinoma, induction chemotherapy, testicular cancer

## Abstract

**Introduction:**

We report a case of advanced testicular cancer cured by early and appropriate resumption of chemotherapy even after COVID‐19 infection during induction chemotherapy.

**Case presentation:**

The patient was a healthy 36‐year‐old male. The diagnosis was a stage IIIB nonseminoma (pT2N2M1a). On day 14 of the first chemotherapy cycle, the patient was diagnosed with mild COVID‐19. The second chemotherapy cycle was initiated with a 1‐day delay (on day 10 after the COVID‐19 diagnosis). The patient achieved remission with minimal postponement of chemotherapy.

**Conclusion:**

Only a few case reports have described the resumption of anticancer chemotherapy in patients with COVID‐19. In deciding when to resume chemotherapy after COVID‐19 infection, it is essential to consider factors such as cancer type, progression, and severity of COVID‐19 and should be tailored to individual patient needs.

**Abbreviations & Acronyms**
BEP: Bleomycin+etoposide+cisplatin
CR: complete response
CT: computed tomography
FEC: Fluorouracil+epirubicin+cyclophosphamide
GEM+nab‐PAC: Gemcitabine+nab‐Paclitaxel
IGCCC: International Germ Cell Classification
NCCN: National Comprehensive Cancer Network
RPLND: retroperitoneal lymph node dissection
TIP: Paclitaxel+ifosphamide+cisplatin

**Keynote message**

We encountered a patient with an advanced testicular tumor and COVID‐19 who went into remission during induction chemotherapy. The timing of chemotherapy resumption should be discussed on a case‐by‐case basis, considering the cancer status, risk of COVID‐19 exacerbation, and significance of chemotherapy.

## Introduction

In 2019, severe acute respiratory syndrome coronavirus 2 (COVID‐19) was first reported in Wuhan, China, followed by a global pandemic. The management of patients with cancer and COVID‐19 infections requires more attention due to the complications of COVID‐19 while commencing cancer treatment. While there are reports that one of the mortality risks for patients with cancer is COVID‐19 infection, the combination with cancer treatment might be controversial given the impact on overall survival due to delays in cancer treatment.^1^, ^2^ Testicular tumors are common malignancies in young males and are one of the most curable malignancies through appropriate multidisciplinary treatment.^3^ This means that managing COVID‐19 infections in patients with testicular tumors is even more important, as treatment delays could affect the disease's cure rate. We report a case of advanced testicular tumor cured by early and appropriate resumption of chemotherapy after being affected by COVID‐19 during induction chemotherapy. In the clinical setting, decision‐making for planning treatment after COVID‐19 during intervention for malignancies is crucial. Thus, we also described the current recommended management map and guidance for chemotherapy plans in this COVID‐19 era.

## Case presentation

**Fig. 1:**
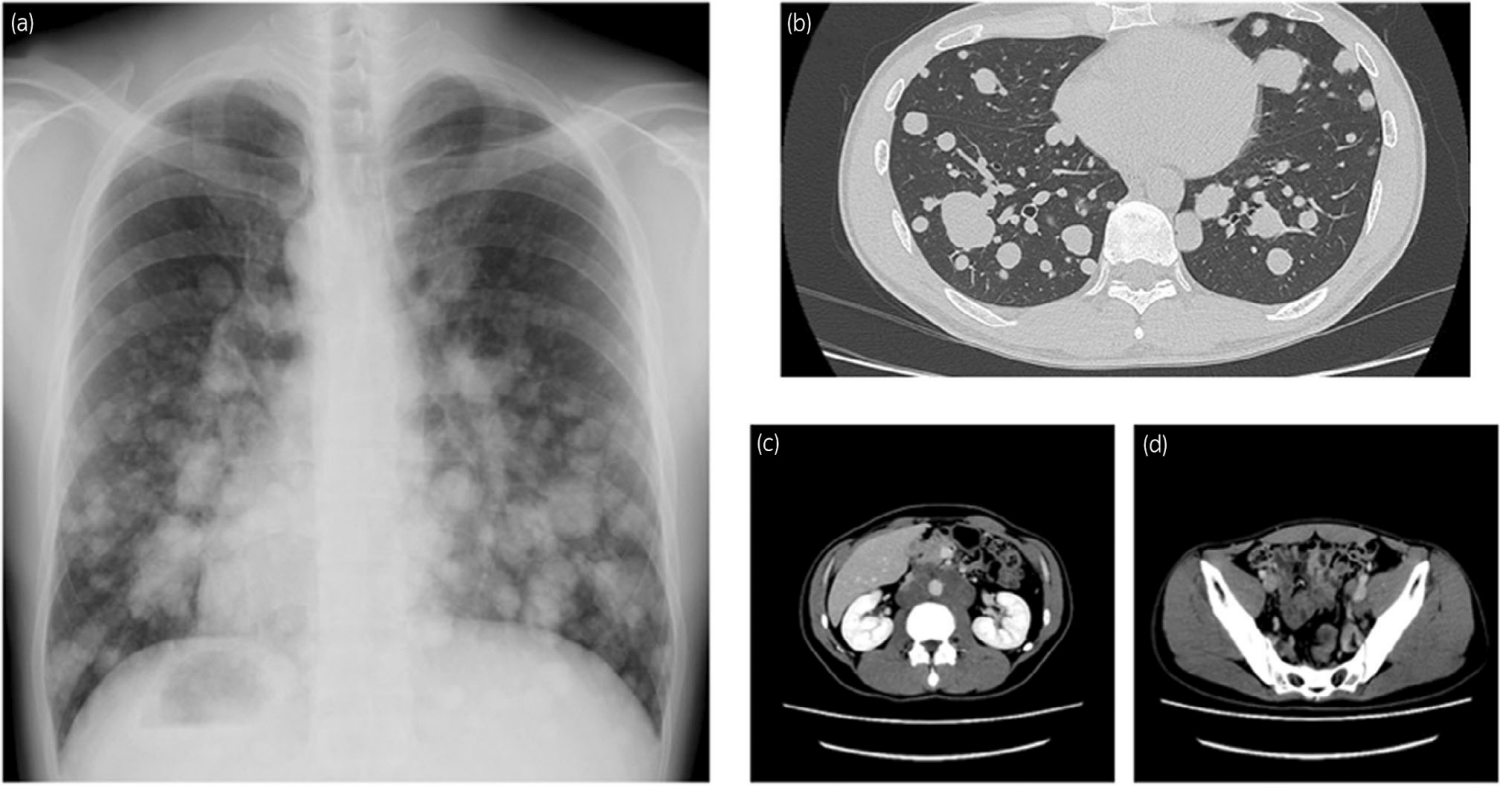
Before BEP, radiography and CT imaging findings. (a) Chest radiography: multiple nodules in both lungs. (b) Multiple lung metastases. (c) Hilar lymph and paravalvular lymph node metastases. (d) Left pelvic lymph node metastases.

**Fig. 2:**
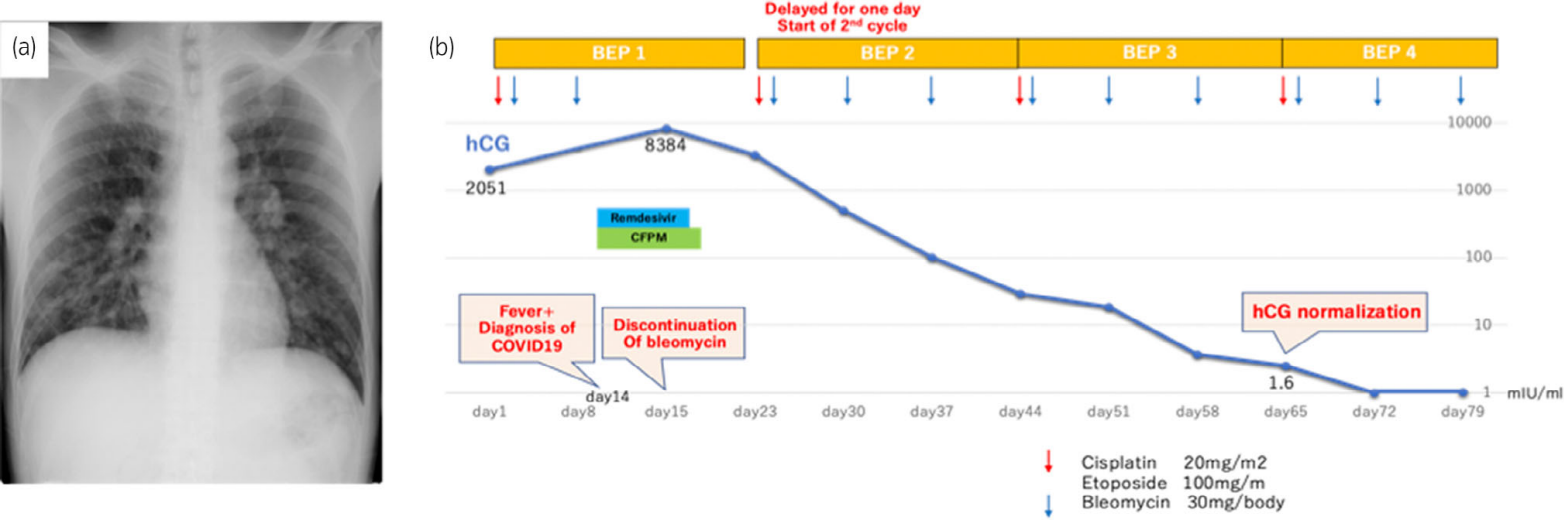
(a) Chest radiography at the time of COVID‐19 diagnosis. (b) Serum hCG levels across the patient's treatment course after anticancer chemotherapy (BEP).

**Fig. 3:**
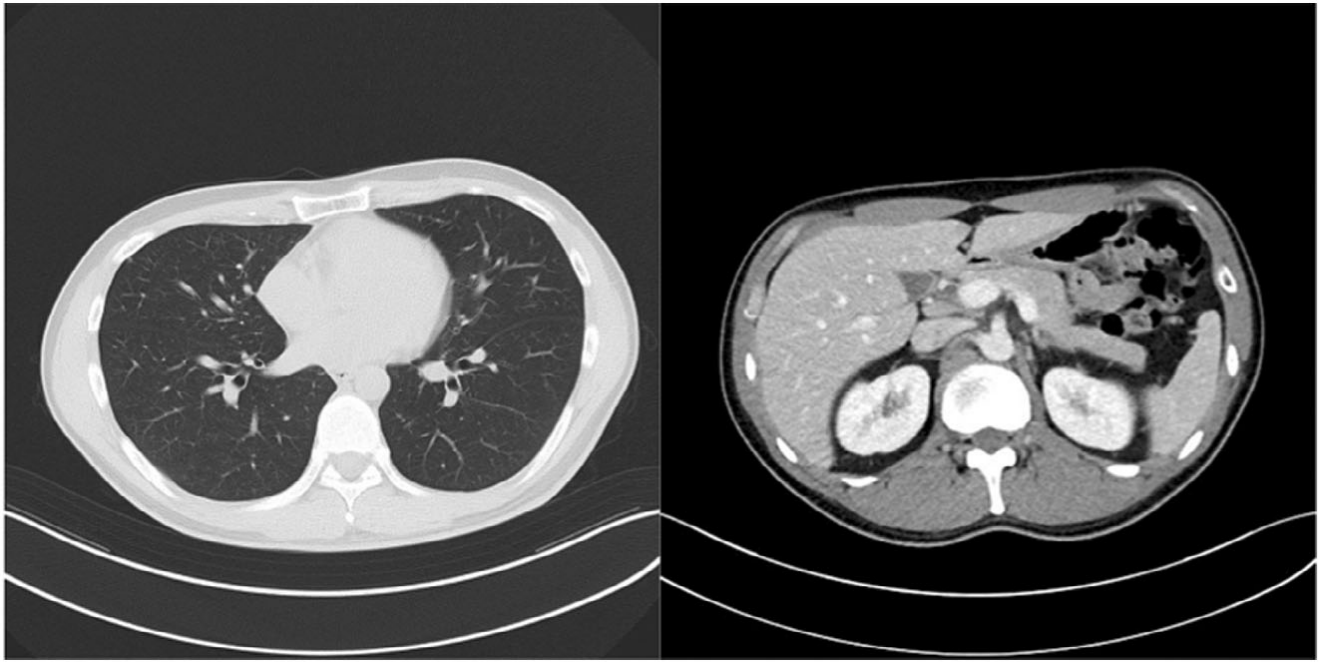
CT imaging showed CR at cycle 4 and subsequent evaluations.

The patient was a healthy 36‐year‐old male. He smoked 20 cigarettes per day for 15 years, was married, and had one child. He had received 3 doses of coronavirus vaccine. In November 2022, he visited his local doctor with the chief complaint of left cervical swelling that he had been aware of for several days. He had a painless enlarged right scrotum, and CT showed multiple lung tumors and enlarged lymph nodes, which led to the suspicion of advanced testicular cancer (Fig. 1). After a right high orchiectomy, the histopathological diagnosis was embryonal carcinoma and seminoma without teratoma. The stage diagnosis was pT2N2M1a, stage IIIB, LDH368U/I, hCG2051mIU/mL, AFP5.3 ng/mL, with an intermediate risk on the IGCCC. On postoperative day 3, the patient started a chemotherapy regimen of BEP composed of cisplatin 20 mg/m^2^ on days 1–5, etoposide 100 mg/m^2^ on days 1–5, and bleomycin 30 mg/body on days 1, 8, and 15 at 3‐week intervals. On day 14 of the first cycle, fever with a body temperature of 38.3°C and cough were observed. Blood tests revealed a white blood cell count of 1740/mm^3^, of which 49.4% were neutrophils and 41.4% were lymphocytes. Chest radiography and CT showed no evidence of pneumonia, and the lung metastases were smaller than before the BEP. The SARS‐CoV‐2 antigen test was positive, and the patient was diagnosed with mild COVID‐19. On the same day, Remdesivir (200 mg loading dose day 14, 100 mg once daily for days 15, 16) and Cefepime (4 g for days 14–19) were initiated because febrile neutropenia due to bacterial infection could not be ruled out. The COVID‐19 symptoms resolved the next day, and the patient's respiratory status remained stable. Bleomycin was discontinued on day 15. The resumption of anticancer chemotherapy was planned after consultation with multiple medical departments, and the second cycle of chemotherapy could be started with a 1‐day delay (on day 10 after the COVID‐19 diagnosis). After the second cycle, the patient progressed smoothly without delay, and the serum hCG level was negative on day 1 of the fourth cycle (Fig. 2). Evaluation after the fourth cycle showed a CR, and no additional chemotherapy or RPLND was necessary. The patient maintained remission of the testicular tumor (Fig. 3).

## Discussion

To the best of our knowledge, there have been only a few case reports of the resumption of anticancer chemotherapy in patients after COVID‐19 infection^4^, ^5^, ^6^, ^7^ (Table 1). In these reports, the timing of chemotherapy resumption varied, which should be considered on a case‐by‐case basis, considering various factors such as the type and progression of cancer and the severity of COVID‐19 infection. As noted earlier, cancer patients are at risk of severe COVID‐19 infection.^1^, ^2^ Regarding anticancer chemotherapy, a meta‐analysis showed an increased risk of COVID‐19 death along with anticancer chemotherapy during the COVID‐19 infection period and 4 weeks prior to infection.^8^ On the other hand, in a prospective cohort study, anticancer chemotherapy in the preceding 4 weeks had no significant effect on COVID‐19‐induced mortality.^9^ Patients resuming anticancer chemotherapy may have an increased risk of COVID‐19 mortality, and caution should be exercised regarding COVID‐19 severity. The NCCN guidelines state that the duration of delay in cancer‐directed therapy is determined on a patient‐by‐patient basis, and if cancer‐directed therapy is urgently required due to uncontrolled cancer, it should be administered at the discretion of the oncologist. It also recommends that patients with mild to moderate COVID‐19 or asymptomatic positive SARS‐CoV‐2 with pulmonary involvement delay therapy for at least 10 days until improvement of symptoms and at least 24 h have passed since resolution of fever without the use of antipyretics.^10^ To ensure cure in induction chemotherapy for advanced testicular tumors, deferral should be considered only if the patient is febrile on the scheduled start date or if blood tests show neutrophils <500/mm^3^ and platelets <100 000/mm^3^, and deferral should be limited to 3 days or less.^11^ In the present case, the start of the second cycle of BEP was limited to a delay of 1 day. In addition, the prognostic impact of skipping bleomycin on day 15 was judged to be low, as 3 cycles of BEP or 4 cycles of EP could be considered for patients with only on LDH 1.5–3 times the upper limit of normal in the IGCCC classification of intermediate risk.^12^ From the above, we have experienced advanced testicular tumor that were able to achieve remission by only requiring minimal postponement of anticancer chemotherapy. As the number of cases in which anticancer chemotherapy is resumed after the COVID‐19 infection increases, the appropriate timing of resumption for each patient needs to be further investigated.

**Table 1:** List of case reports

Author	Cancer type	Age	Sex	Stage	Year of COVID‐19 infection	Severity of COVID‐19	Days from diagnosis of COVID‐19 to restart of chemotherapy	Regimen
Horiguchi *et al*.^4^	Breast cancer	38	F	IIB	2020	Moderate	45 days	FEC
Nagai *et al*.^5^	Pancreatic cancer	67	M	III	2020	Moderate	49 days	GEM+nab‐PAC
Liontos *et al*.^6^	Ovarian cancer	60	F	IIIc	–	Severe	27 days	Weekly PAC
Tanabe *et al*.^7^	Mediastinal tumor non seminoma	18	M	–	2021	Mild	16 days	BEP
Saito *et al*. (JSMO2023; P53‐6)	Testicular tumor non seminoma	38	M	–	–	Mild	4 days	TIP
This case	Testicular tumor non seminoma	36	M	IIIB	2022	Mild	10 days	BEP

## Conclusion

This patient with an advanced testicular tumor achieved remission with minimal postponement of anticancer chemotherapy. Consideration of factors such as cancer type, progression, and COVID‐19 severity is essential to determine the timing of chemotherapy resumption after COVID‐19. Caution should be exercised regarding COVID‐19 severity, and treatment decisions should be tailored to individual patient needs.

## Author contributions

Goshi Kitano: Conceptualization; data curation; writing – original draft. Shiori Tanaka: Data curation. Manabu Kato: Data curation. Naoya Itoh: Supervision; writing – review and editing. Takahiro Kojima: Supervision; writing – review and editing.

## Conflict of interest

The authors declare no conflict of interest.

## Approval of the research protocol by an Institutional Reviewer Board

Not applicable.

## Informed consent

Written informed consent was obtained from the patient for publication of this report.

## Registry and the Registration No. of the study/trial

Not applicable.
